# Psychometric validation of Young’s Internet Addiction Test among Chinese undergraduate students

**DOI:** 10.1371/journal.pone.0320641

**Published:** 2025-04-24

**Authors:** Zhixia Wei, Norlizah Che Hassan, Siti Aishah Hassan, Normala Ismail, Xiaoxia Gu, Jingyi Dong

**Affiliations:** 1 Hebei Key Laboratory of Children’s Cognition and Digital Education, School of Educational Studies, Langfang Normal University, Langfang, Hebei, China; 2 Faculty of Educational Studies, Universiti Putra Malaysia, Serdang, Selangor, Malaysia; Faculty of Medicine and Pharmacy of Agadir, MOROCCO

## Abstract

The global prevalence of internet addiction is escalating annually and uncontrollable use of the internet can cause significant physical and psychological damage. Young’s Internet Addiction Test (IAT), widely utilized across diverse cultures, has demonstrated structural inconsistencies in previous research, with some items requiring refinement. This study aimed to validate the IAT among Chinese undergraduate students and assess its psychometric properties. The IAT’s structure was initially explored through Exploratory Factor Analysis (EFA) using pilot study data (n=376), with internal consistency and test-retest reliability (n=96) evaluated. Subsequently, Confirmatory Factor Analysis (CFA) was conducted using data from the actual study (n=1042) to confirm the structure. Results showed that a three-factor solution explained 61.29% of the total variance with a satisfactory model fit (χ^2^/df = 4.382, RMSEA = 0.057, CFI = 0.952, TLI = 0.943, SRMR = 0.045, AIC = 798.755) and psychometric properties, validating the IAT’s utility for future investigations of internet addiction in Chinese undergraduates. Notably, a high prevalence of moderate internet addiction was observed within the sample, highlighting the significance of this issue in the target population and emphasizing the need for further research and potential interventions.

## 1. Introduction

With the widespread of the internet, the global prevalence of internet addiction has been approximately 14.22% in the past 20 years and this number is increasing year by year [1]. Besides that, China has the greatest rate of internet addiction among undergraduate students when compared to Japan, Singapore, and the US [2]. Uncontrollable use of the internet can cause significant physical and psychological damage [3,4]. Moreover, internet addiction has a negative impact on the academic performance of students because they spend too much time on internet entertainment, which makes them less engaged in their academics and insensitive to outside voices, including the speech of teachers [2,5]. As a result, finding a solution to the internet addiction problem among students has become critical now. Correspondingly, the issues of cross-cultural consistency and validity of internet addiction measurement instruments have become increasingly prominent. The assessment of internet addiction predominantly relies on self-report questionnaires as the primary measurement method. However, different scales and diagnostic criteria can lead to variations in students’ detection rates of internet addiction [6–8]. Therefore, reliable and consistent instruments will not only facilitate the diagnosis of internet addiction but also make the cross-cultural comparisons of it easier.

The Internet addiction test (IAT) developed by Young in 1998 is one of the most widely used internet addiction measurement instruments across different cultures [9]. Although there was a lack of systematic examination when it was published, it has been validated in different cultural contexts and has shown good psychometric properties [8]. However, its structure has shown great inconsistency in previous studies and there is no consensus yet. The validation of the IAT across diverse cultural contexts has yielded heterogeneous factorial structures, ranging from single-factor to six-factor solutions [10–15]. This variability in factorial solutions may be attributed to differences in respondent demographics, linguistic backgrounds, cultural contexts, and statistical methodologies employed [16].

In the Chinese context, IAT validation studies have predominantly focused on Hong Kong populations and discrepancies in factor solutions persist. Chang and Man Law [12] proposed a three-factor solution for Hong Kong undergraduate students, which was subsequently corroborated by Lai et al. [16,17] in samples of Hong Kong Chinese adolescents and secondary school students. Chen and Nath [18] identified a four-factor solution in a sample of Chinese undergraduate students, while Ali et al. [19] developed a unidimensional six-item version of the IAT for Hong Kong school children. More recently, Lu et al. [20] proposed a three-factor solution based on college students from Zhejiang province. Notably, to the best of our knowledge, there is a lack of validation studies focusing on normal undergraduate students in China, who are individuals trained at normal Universities to become primary, secondary, or kindergarten teachers [21]. This study aims to address this gap by providing empirical evidence on the factor structure of the IAT in this previously unexplored population, thereby contributing to the ongoing discourse on the scale’s factorial composition.

Given that nearly three decades have elapsed since the IAT was compiled, several items have been identified as outdated or unsuitable for assessing undergraduate students [11,20,22–24]. Consequently, there is a need to update or reformulate certain items’ expressions or translations. Furthermore, as the original IAT development process did not adhere to the standard procedure of conducting exploratory factor analysis (EFA) followed by confirmatory factor analysis (CFA), this study aims to explore the IAT’s factor structure using this methodologically rigorous approach. In addition, the significance of investigating internet addiction among normal undergraduate students extends beyond their individual well-being and academic achievements, as it has implications for the quality of education that future students will receive. However, despite the importance of this population, there is a relative paucity of research utilizing the IAT to assess internet addiction levels among Chinese normal undergraduate students.

In light of these considerations, this study seeks to address the aforementioned research gaps by validating the IAT in a sample of Chinese normal undergraduate students, employing a comprehensive methodological approach to elucidate its factor structure and psychometric properties within this specific cultural and educational context. To be more specific, this study aimed to: (1) update the items of the IAT and rephrase the items not suitable for the population of undergraduate students; (2) explore the structure of the IAT through Exploratory Factor Analysis (EFA) using the data of pilot study and evaluate the internal consistency reliability and test-retest reliability of IAT; (3) confirm its structure with Confirmatory Factor Analysis (CFA) using the data of actual study and evaluate the psychometric properties of IAT including its construct validity, convergent validity, composite reliability, discriminant validity and criterion validity; (4) identify the internet addiction level of the sampled normal undergraduate students.

## 2. Methods

### 2.1. Study design

This investigation employed a cross-sectional, quantitative research design implemented over a five-week period from June 5 to July 10, 2023. The study protocol followed a systematic three-phase structure, with each phase addressing specific psychometric objectives. The initial phase encompassed a pilot study for EFA, followed by a second phase dedicated to examining test-retest reliability. The final phase was devoted to CFA to validate the factor structure of the adapted IAT.

### 2.2. Population

The present study employed a multi-phase sampling design drawing from undergraduate students enrolled in normal universities across H Province, China—a region with six normal universities and a long history of teacher education. From these six universities, four were randomly selected to constitute the sampling frame. According to the official institutional data, the total undergraduate student population across these normal universities was approximately 64,393. The student population distribution among the selected universities was 18,958, 19,346, 14,067, and 12,022 students, respectively. Participant eligibility criteria specified current enrollment as an undergraduate student at one of the four selected universities, while excluding individuals on academic leave or those with documented mental health conditions.

### 2.3. Sample size and procedures

A stratified random sampling approach was implemented to ensure representative participation from each institution, which was chosen to enhance the generalizability of findings across diverse student populations [25]. The determination of appropriate sample sizes followed established methodological guidelines for factor analysis. A subject-to-variable ratio of 10:1 or a minimum of 5:1 is recommended for EFA [26]. Given that the IAT comprises 20 items, a minimum sample size of 200 participants was deemed necessary for the pilot phase of this study. Regarding CFA, the literature suggests a range of 5–20 participants per item for validation studies [27,28]. Adopting a conservative approach and utilizing the upper bound of this recommendation, a sample size exceeding 400 participants was targeted for the CFA phase.

To account for potential attrition and non-response, a 50% oversample was incorporated into the calculations, necessitating additional samples of 100 participants for the pilot study (EFA phase) and 200 for the CFA phase. Consequently, the final target sample sizes were set at 300 and 600 participants for the pilot and CFA phases, respectively. Employing stratified sampling methodology, the sample allocation across institutions was calculated using the formula: Sample = (size of entire sample)/ (population size) × layer size. This resulted in required minimum sample sizes for the pilot study of 88, 90, 66, and 56 students from the respective institutions, while the CFA phase required 177, 180, 131, and 112 participants. The final participant selection was executed using the random sampling function in SPSS version 26.0, utilizing student matriculation numbers as the sampling frame.

### 2.4. Ethical approval

This research received ethical approval from the Ethics Committee for Research involving Human Subjects of University Putra Malaysia Research (NO: JKEUPM-2023–137) and the written informed consents of participants were obtained before their involvement. In the whole testing process, if the participants are unwilling to cooperate, they can withdraw from the study at any time.

### 2.5. Participants

In the first phase of the study (pilot study), a sample of 383 participants was used to detect potential problems and seven participants were excluded from the further analysis after data examination with an effective rate of 98.17% (55.32% females, mean age=20±1.63 years). In the second phase, test-retest reliability was analyzed using one sample of 98 participants who had also attended the pilot study and two questionnaires were removed after data screening with an effective rate of 97.96% (63.54% females, mean age=19±0.86 years). In the third phase, a total of 1056 participants were involved in the study and a sample of 1042 was used for confirmation factor analysis (CFA) after the data examination with an effective rate of 98.67% (59.98% females, mean age=20±1.95 years).

### 2.6. Measures

#### Internet Addiction Test (IAT).

IAT was developed by Young [29,30], a 20-item self-report instrument designed to assess symptoms of problematic internet use. Respondents rate the frequency of behaviors and feelings associated with internet use on a 5-point Likert scale, ranging from 1 (rarely) to 5 (always). The IAT yields a total score between 20 and 100, with higher scores indicative of more severe internet addiction symptomatology. The IAT employs established cut-off scores to stratify individuals into four distinct categories of internet addiction severity: scores below 30 indicate absence of addiction; 31–49 suggest mild addiction; 50–79 denote moderate addiction; and 80–100 signify severe addiction. While there is some variation, a score of 50 on the IAT is the most widely accepted cut-off point for determining the prevalence of internet addiction in current literature [9,15,23,24,29–31]. In the present study, the adapted IAT demonstrated robust psychometric properties, exhibiting good reliability and validity.

#### Internet addiction test adaptation.

The IAT underwent a rigorous translation process involving forward and backward procedures [32]. Moreover, several items were modified to better reflect the contemporary internet usage patterns of undergraduate students and to address concerns raised in recent literature. The details of the modifications were as follows: “Household chores” in item 2 was replaced with “daily routines, such as bathing and eating” to align with campus life [33]. “Intimacy with your partner” of item 3 was changed to “spending time with a close person” to accommodate students without romantic partners [11,17,24,34]. Item 6: “Work” was modified to “schoolwork/grades” to better reflect the student context [15,20,34–36]. “Email” in item 7 was updated to “online instant message (e.g., QQ, WeChat)” to account for evolving communication technologies and the context of the vast majority of respondents in this study rarely use email [22,23] and check email may no longer serve as a reliable indicator of problematic internet use [14,20,23,24,31,34,37]. “Job performance” of item 8 was replaced with “academic performance” to suit the student sample [15,35,36,38]. Item 14: “Do you lose sleep due to being online?” was clarified to “Do you have sleep deprivation due to being online?” to address ambiguity and improve factor loading [29,39]. “Going out with others” of item 19 was revised to “doing previously enjoyed hobbies and/or outside interests” to avoid redundancy with Item 3 and broaden the scope of off-line activities [24,33,40–42]. The rephrased items are presented in Table 2, denoted by italics.

**Table 2:** Item analysis for IAT (n=1042).

No.	Items (How often…)	M±SD	Skewness	Kurtosis
1	Do you find that you stay online longer than you intended?	3.10±1.07	0.02	‒0.77
2	*Do you neglect daily routines such as bathing and eating because of surfing the Internet?*	2.64±1.00	0.45	‒0.14
3	*Do you prefer the excitement of the Internet to spending time with a close person?*	2.20±1.01	0.74	0.36
4	Do you form new relationships with fellow online users?	1.88±0.97	1.26	1.36
5	Do others in your life complain to you about the amount of time you spend online?	2.48±1.04	0.40	‒0.38
6	*Do your grades or school work suffer because of the amount of time you spend online?*	2.45±1.01	0.45	‒0.20
7	*Do you check your online instant message (e.g., QQ, WeChat) before something else that you need to do?*	1.79±0.94	1.20	1.13
8	*Does your academic performance or productivity suffer because of the Internet?*	2.38±1.10	0.50	‒0.44
9	Do you become defensive or secretive when anyone asks you what you do online?	3.01±1.17	0.12	‒0.88
10	Do you block out disturbing thoughts about your life with soothing thoughts of the Internet?	2.85±1.18	0.09	‒0.88
11	Do you find yourself anticipating when you will go online again?	2.51±1.17	0.41	‒0.68
12	Do you fear that life without the Internet would be boring, empty, and joyless?	2.80±1.16	0.17	‒0.78
13	Do you snap, yell, or act annoyed if someone bothers you while you are online?	1.85±0.94	1.04	0.80
14	*Do you have sleep deprivation due to being online?*	2.47±1.12	0.45	‒0.54
15	Do you feel preoccupied with the Internet when off-line, or fantasize about being online?	2.40±0.98	0.52	‒0.03
16	Do you find yourself saying “just a few more minutes” when online?	2.61±1.12	0.27	‒0.72
17	Do you try to cut down the amount of time you spend online and fail?	2.35±1.06	0.50	‒0.44
18	Do you try to hide how long you’ve been online?	1.87±0.98	1.03	0.60
19	*Do you choose to spend more time online over doing previously enjoyed hobbies and/ or outside interests?*	1.85±0.97	1.07	0.69
20	Do you feel depressed, moody, or nervous when you are off-line, which goes away once you are back online?	1.75±0.94	1.19	0.92

#### Content validity and face validity of the adapted IAT.

The content validity and face validity of the adapted IAT were examined using the expert judgment method and three psychological professors were invited to evaluate the conformity of the questionnaire’s items to the original content range. Feedback from the panel of experts indicates that the items effectively capture the topic under investigation, they represent the intended content area based on the objectives of the study very well, and there are no common errors such as wrong spelling, blurred instructions, or confusing questions. The content validity and face validity of the questionnaires meet the requirements. According to the feedback from the respondents during the pilot study, all the instructions and the items of the scale can be understood clearly.

#### Demographics, internet use information, and academic performance.

Data were collected on socio-demographic variables such as age, gender, major and academic year, and internet use profiles including average hours spent online every day for leisure without relation to work or study, online activities, and internet usage years [12,23,30,34]. Academic performance was assessed using a single-item self-report measure. Participants responded to the question “How is your academic performance?” on a 5-point Likert scale [12,24]. The response options ranged from 1 (low) to 5 (excellent), with intermediate points representing fair (2), good (3), and very good (4) performance. Higher scores on this scale were indicative of better perceived academic performance.

### 2.7. Data collection

The adaptation test was conducted firstly to ensure that the content validity and face validity of the scale are suitable for the population of Chinese undergraduate students. Then, the electronic questionnaires were distributed through the Questionnaire Star platform for a pilot study to examine the consistency of the items and the understanding of the participants to the questions. The students can fill the questionnaires using WeChat and the construct of the IAT was analyzed based on the data of pilot study using EFA. After two weeks, a retest was carried out among 98 participants of the pilot study to assess the test-retest reliability. The intervals between repeated measurements and the sample size were consistent with previous studies [11,22,24,43–46]. Finally, the questionnaires were distributed to undergraduate students from four normal universities who did not attend this study previously and the data was used for CFA and the internet addiction level of the participants was identified.

### 2.8. Statistical analysis

After data collection, SPSS 26.0 was used for data examination, descriptive statistical analysis and EFA, and the CFA was performed with Amos 24.0 using Maximum Likelihood Estimation.

The EFA was used to extract factors and determine psychometric constructs for the IAT based on the pilot data. The Principal Component Analysis (PCA) with orthogonal rotation (varimax) method was adopted to extract factors, which gets out factors based on the correlation among the items, and it is the most commonly used extraction technique [47]. Bartlett’s test of sphericity [48], and the Kaiser-Meyer-Olkin (KMO) measure of sampling adequacy [49] were used to assess the factorability of the data. Bartlett’s test of sphericity should be significant (p <0.05) and the KMO values should above 0.6 for factor analysis [50,51]. Three techniques including Kaiser’s criterion, scree test, and parallel analysis were used to decide the number of factors to retain. Based on Kaiser’s criterion, only factors with an eigenvalue of 1.0 or more are remined for further analysis [49]. According to Scree test, all factors above the elbow in the plot should be retained because these factors explain the most of the data variance [52]. Horn’s parallel analysis compares the size of the eigenvalues with eigenvalues produced by a randomly generated dataset of the same size and only the eigenvalues exceed the corresponding values of the random data set are considered to retain [53]. Cronbach’s alpha coefficients and intraclass correlation coefficients (ICCs) were used to check the internal consistency and the test-retest reliability of the IAT [54].

CFA was employed to validate the measurement model by evaluating the significance of individual items within the construct. Items with factor loadings below 0.5 were considered to have minimal contribution to the measurement construct and were candidates for elimination [26]. Model fit was assessed using a combination of Root Mean Square Error of Approximation (RMSEA), Comparative Fit Index (CFI), Tucker-Lewis Index (TLI), χ^2^/df, and Standardized Root Mean Square Residual (SRMR) for fit assessment [55]. Acceptable thresholds were set as follows: RMSEA < 0.10 (preferably < 0.08), CFI and TLI > 0.90, χ^2^/df < 5.0 [51], and SRMR ≤ 0.08 [56]. Model refinement involved eliminating problematic items or correlating error covariances based on Modification Indices exceeding 15 [57]. Convergent validity was evaluated using Average Variance Extracted (AVE), with values > 0.50 considered acceptable [58]. Composite Reliability (CR) was calculated for sub-constructs, with a threshold of > 0.7 [26]. Discriminant validity was assessed using the AVE-SV approach, requiring the square root of AVE to exceed corresponding correlations [58], and inter-construct correlations to remain below 0.85 [59].

Pearson correlation coefficients were computed to examine relationships between internet addiction and criterion variables. The prevalence and severity of internet addiction among the sampled students were determined through descriptive statistical analyses.

## 3. Results

### 3.1. Descriptive analysis

Demographic data were collected from participants, encompassing variables such as institution, gender, academic year, field of study, family residence, single-parent family status, and socioeconomic background. A test-retest reliability assessment was conducted with a subset of participants from the first and second academic years at one of the participating universities. Table 1 presents the frequency distributions and percentages of participants across the three phases of the study, stratified by various demographic characteristics.

**Table 1:** Participants distribution on demographic variables.

Demographic Variables		Pilot study(n=376) n (%)	Retest study (n=96) n (%)	Validation study (n=1042) n (%)
University	1	105(27.92)	0(0.00)	238(22.84)
	2	96(25.53)	96(100.00)	356(34.17)
	3	89(23.67)	0(0.00)	268(25.72)
	4	86(22.87)	0(0.00)	180(17.27)
Gender	Male	168(44.68)	35(36.46)	417(40.02)
	Female	208(55.32)	61(63.54)	625(59.98)
Academic Year	First Year	112(29.79)	49(51.04)	293(28.12)
	Second Year	97(25.80)	47(48.96)	287(27.54)
	Third Year	89(23.67)	0(0.00)	249(23.90)
	Fourth Year	78(20.74)	0(0.00)	213(20.44)
Major	Humanities	142(37.77)	49(51.04)	410(39.35)
	Science & Engineering	118(31.38)	47(48.96)	318(30.52)
	Arts	116(30.85)	0(0.00)	314(30.13)
Family Location	Rural	221(58.78)	58(60.42)	632(60.65)
	City	155(41.22)	38(39.58)	410(39.35)
Single-Parent Family	Yes	27(7.18)	3(3.12)	80(76.78)
	No	349(92.82)	93(96.88)	962(92.32)
Family Financial Situation	Wealthy	45(11.97)	12(12.50)	133(12.76)
	Average	263(69.95)	67(69.79)	750(71.98)
	Poor	68(18.08)	17(17.71)	159(15.26)

In the validation phase of the study, supplementary data were collected regarding participants’ internet usage patterns and academic performance. Analysis revealed that the mean daily online time was 5.47 hours (SD = 2.17), while the average duration of internet use experience was 7.39 years (SD = 2.36). Regarding online activities, the largest number of participants chose to watch videos (88.67%), followed closely by online chatting (87.33%). Online shopping (71.33%) and gaming (59.33%) also constituted significant portions of online activities. Notably, the lowest number of participants chose to browse the web (51.33%).

Item analysis results for the IAT are presented in Table 2. The mean scores for individual items ranged from 1.75 (item 20) to 3.10 (item 1), with standard deviations approximating 1 across items. Skewness values fell within the range of 0.02 to 1.26, while kurtosis values ranged from -0.88 to 1.36. These metrics suggest that the data conform to a normal distribution, adhering to the commonly accepted thresholds for univariate normality in psychometric research [51]. This normality in item responses supports the use of parametric statistical techniques in subsequent analyses and suggests that the scale is capable of capturing a range of internet addiction severity levels in the target population [55,60].

### 3.2. Exploratory factor analysis

The KMO measure of sampling adequacy for IAT yielded a value of 0.93, exceeding the recommended minimum threshold of 0.6. Additionally, Bartlett’s Test of Sphericity was significant (χ^2^ (190) = 4305.01, p <.001). Consequently, the IAT comprising 20 items was suitable for conducting EFA.

Table 3 displays the total variance explained by the components extracted through PCA method. The result indicates that PCA procedure has extracted three dimensions with eigenvalues over Kaiser’s criterion of 1 (8.88, 1.92, 1.47). Collectively, these three components account for 61.29% of the total variance. Specifically, Factor 1 explains 44.38% of the variance, indicating it is the most significant factor, while Factor 2 accounts for 9.58% and Factor 3 contributes 7.33% to the total variance.

**Table 3:** Total variance explained by IAT.

Component	Initial Eigenvalues			Extraction Sums of Squared Loadings			Rotation Sums of Squared Loadings		
	Total	% of Variance	Cumulative %	Total	% of Variance	Cumulative %	Total	% of Variance	Cumulative %
1	8.88	44.38	44.38	8.88	44.38	44.38	4.39	21.93	21.93
2	1.92	9.58	53.96	1.92	9.58	53.96	4.35	21.73	43.67
3	1.47	7.33	61.29	1.47	7.33	61.29	3.53	17.63	61.29
4	0.85	4.23	65.52						
5	0.77	3.86	69.38						
6	0.71	3.56	72.93						

Extraction Method: Principal Component Analysis.

**Fig 1:**
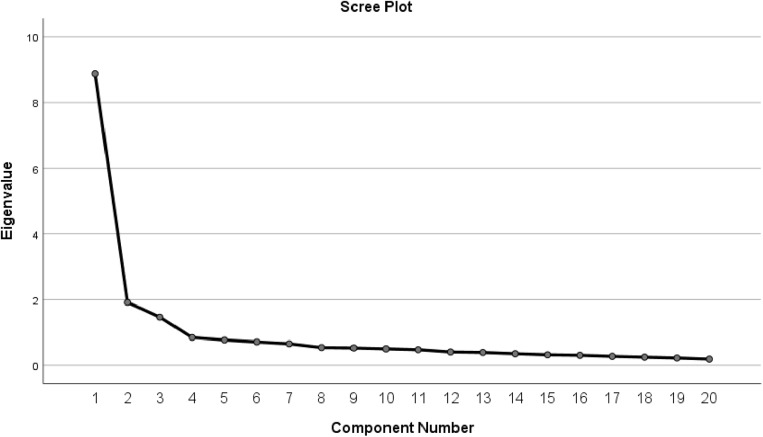
Scree plot of IAT.

The Scree Plot of the IAT, illustrated in Fig 1, reveals that there is a distinct inflexion point following the third factor, which indicates factors 1, 2, and 3, especially factors 1 and 2, capture much more of the variance than the remaining factors. Additionally, the proportion of total variance explained by the subsequent factors progressively diminishes starting from the third factor.

Parallel analysis employing the Monte Carlo PCA program constitutes the third method for determining the appropriate number of factors to retain. As presented in Table 4, a comparison is made between the eigenvalues derived from PCA and the criterion eigenvalues obtained through parallel analysis. This comparison indicates that three factors possess eigenvalues exceeding the corresponding criterion eigenvalues from a simulated data matrix of the same size (20 variables × 376 respondents) with 1000 iterations. Specifically, the eigenvalue of the first factor in the PCA results is 8.88, whereas the criterion eigenvalue from the parallel analysis is 1.42. For the second factor, the PCA eigenvalue is 1.92 compared to 1.35 in the simulation data. Additionally, the third factor has an eigenvalue of 1.47 in the actual data, which surpasses the criterion eigenvalue of 1.29 in the simulated data. Consequently, parallel analysis supports the retention of all three factors, endorsing a three-factor solution for the IAT.

**Table 4:** Comparison of eigenvalues from PCA and parallel analysis.

Component	Actual Eigenvalue from PCA	Criterion Value from Parallel Analysis	Decision
1	8.88	1.42	Accept
2	1.92	1.35	Accept
3	1.47	1.29	Accept

The Rotated Component Matrix displays the factor loadings for each item across the three components generated by SPSS, with eigenvalues exceeding 1, as derived from PCA. Table 5 delineates the factor loadings following varimax rotation, demonstrating that all items exhibit strong loadings (exceeding 0.5) on the three factors. Furthermore, no item shows cross-loadings above 0.20 on two or more factors, suggesting that the three-factor model is most suitable. This finding reinforces the decision to maintain three factors for subsequent analysis, consistent with the results from parallel analysis.

**Table 5:** EFA from the varimax rotation for IAT (n=376).

						Rotated Factor Loading		
**Item**	**M±SD**	**Skewness**	**Kurtosis**	**Corrected item-total correlation**	**Alpha without item**	**WSP**	**ECP**	**LCI**
1	2.91±0.98	0.00	‒0.68	0.560	0.930			0.632
2	2.49±0.89	0.35	‒0.08	0.678	0.927			0.639
3	1.95±0.85	0.65	0.17	0.540	0.930	0.547		
4	2.23±0.82	0.32	‒0.04	0.482	0.931	0.650		
5	2.40±0.89	0.40	‒0.16	0.560	0.930			0.511
6	2.35±0.88	0.36	0.01	0.648	0.928			0.823
7	1.83±0.85	0.83	0.29	0.630	0.928	0.786		
8	2.29±0.91	0.35	‒0.23	0.657	0.928			0.679
9	2.81±1.00	0.38	‒0.21	0.496	0.931			0.753
10	2.65±1.08	0.21	‒0.63	0.561	0.930		0.660	
11	2.30±1.00	0.43	‒0.55	0.672	0.927		0.752	
12	2.48±1.08	0.38	‒0.57	0.617	0.929		0.768	
13	1.77±0.82	0.72	‒0.29	0.676	0.928	0.673		
14	2.15±1.00	0.70	0.07	0.625	0.928		0.620	
15	2.26±0.91	0.60	0.21	0.735	0.926		0.712	
16	2.46±0.94	0.20	‒0.68	0.661	0.928		0.725	
17	2.25±0.91	0.29	‒0.53	0.706	0.927		0.616	
18	1.77±0.84	0.84	‒0.10	0.597	0.929	0.719		
19	1.65±0.79	1.03	0.55	0.628	0.928	0.809		
20	1.68±0.81	0.93	‒0.02	0.621	0.928	0.780		
Eigenvalue						8.88	1.92	1.47
Explained variance (%)						44.38	9.58	7.33
Cronbach’s Alpha (α)						0.88	0.89	0.85

Table 5 illustrates that Factor 1 comprises seven items, specifically items 3, 4, 7, 13, 18, 19, and 20, which pertain to reliance on the internet for social interactions and interpersonal relationships, as well as emotional and behavioral responses when internet access is unavailable. This factor encompasses many established addiction symptoms, including preoccupation, withdrawal, and social impairment, thereby warranting the designation “Withdrawal and Social Problems (WSP).” Factor 2 consists of seven items—10, 11, 12, 14, 15, 16, and 17—that are best described as “Emotional and Cognitive Preoccupation (ECP)” with the internet, as these items primarily capture the emotional and cognitive reactions of respondents towards internet usage. Lastly, Factor 3, including items 1, 2, 5, 6, 8, and 9, can be termed “Loss of Control and Interference with Daily Life (LCI)” due to its reflection of respondents’ experiences of overuse, loss of control over online activities and desires, and the negative impact on daily life and academic responsibilities.

A positive linear correlation exists among the three factors: Withdrawal and Social Problems (WSP: M = 1.84, SD = 0.63), Emotional and Cognitive Preoccupation (ECP: M = 2.37, SD = 0.77), and Loss of Control and Interference with Daily Life (LCI: M = 2.55, SD = 0.71). Specifically, the correlation coefficients are as follows: between WSP and ECP, 0.61 (p < 0.01, two-tailed); between WSP and LCI, 0.56 (p < 0.01, two-tailed); and between ECP and LCI, 0.66 (p < 0.01, two-tailed). These values are considered acceptable and suggest both independence and interrelation among the factors in accounting for the variance, which supports the validity of the three-factor model of IAT.

### 3.3. Reliability analysis

To assess the internal consistency of the IAT, Cronbach’s alpha coefficient was computed. As outlined in Table 5, the Cronbach’s alpha values for Factor 1 (ECP), Factor 2 (WSP), and Factor 3 (LCI) were 0.88, 0.89, and 0.85, respectively, all exceeding the established threshold of 0.70 [61], indicating strong internal consistency. The reliability analysis of IAT construct, yielded an overall Cronbach’s alpha of 0.932. Additionally, the “Alpha if item deleted” values ranged from 0.927 to 0.931, showing no increase in alpha with the removal of any single item. Furthermore, all item-total correlations were above 0.40. These findings collectively demonstrate the scale’s high level of internal consistency and suggest that no items should be excluded.

The test-retest reliability of the IAT was evaluated using ICCs, which were computed to evaluate the stability of scores over time. Participants (n = 96) completed the scale at two-time points, separated by a 2-week interval. Results indicated good test-retest reliability for the overall IAT (ICC = 0.783, 95% CI [0.691, 0.849]) and its subscales: WSP (ICC = 0.775, 95% CI [0.681, E0.844]), ECP (ICC = 0.851, 95% CI [0.785, 0.898]), and LCI (ICC = 0.827, 95% CI [0.751, 0.881]). These findings suggest that the IAT demonstrates temporal stability, supporting its use as a reliable measure of internet addiction.

### 3.4. Confirmatory factor analysis

CFA was performed to validate the three-factor solution of the IAT identified through EFA. The initial measurement model for the IAT demonstrated that all 20 items had factor loadings exceeding 0.50. Model fit indices (Model 1 in Table 6) revealed that the RMSEA was 0.072 (<0.08), the CFI was 0.921 (>0.90), and the TLI was 0.910 (>0.90), indicating a satisfactory fit. The SRMR was 0.050, which is below the upper limit of 0.080. However, the χ^2^/df was 6.365, exceeding the acceptable threshold of 5.0, thereby not meeting the required model fit criteria.

**Table 6:** Comparison of model fit indices between the initial model and final model.

	χ^2^/df	RMSEA (90% CI)	CFI	TLI	SRMR	AIC
Model 1	6.365	0.072 [0.068-0.076]	0.921	0.910	0.050	1148.947
Model 2	4.382	0.057 [0.053-0.061]	0.952	0.943	0.045	798.755

Examination of modification indices indicates that allowing certain item error variances to covary may enhance model fit, a practice employed in previous research [17,22,24,35,36,42,62]. Guided by these indices, error covariances were correlated between items 1 and 2, both of which load on the LCI factor and assess the degree to which respondents experience diminished control due to internet usage. Similarly, error covariances were specified between items 11 and 12, both of them load on the ECP factor and measure the extent of respondents’ emotional reliance on the internet. Additionally, error covariances were correlated between items 18 and 19, both of which load on the WSP factor and evaluate the social difficulties experienced by respondents as a consequence of internet addiction.

The fit indices for the re-specified measurement model, incorporating error correlations, are presented in Model 2 of Table 6. All items demonstrate factor loadings exceeding the threshold value of 0.5 across their respective dimensions (Table 7). The final measurement model of internet addiction exhibits satisfactory model fit indices (χ^2^/df = 4.382 < 5.0, RMSEA = 0.057 < 0.08, CFI = 0.952 > 0.90, TLI = 0.943 > 0.9). The SRMR value of 0.045 falls below the 0.080 criterion. Furthermore, the Akaike Information Criterion (AIC) for the re-specified model (798.755) is lower than that of the initial model (1148.947), indicating superior fit as lower AIC values are preferable. Consequently, the final model was acceptable, which also demonstrated that the construct validity of IAT can meet the requirement because the fitness indices have achieved the recommended threshold.

**Table 7:** AVE and CR for IAT.

Sub-constructs	Items	Loadings	AVE	CR
WSP	IA3	0.69	0.527	0.885
	IA4	0.56		
	IA7	0.83		
	IA13	0.67		
	IA18	0.76		
	IA19	0.75		
	IA20	0.79		
ECP	IA10	0.70	0.526	0.885
	IA11	0.64		
	IA12	0.72		
	IA14	0.75		
	IA15	0.67		
	IA16	0.85		
	IA17	0.73		
LCI	IA1	0.59	0.531	0.869
	IA2	0.67		
	IA5	0.71		
	IA6	0.86		
	IA8	0.88		
	IA9	0.61		

### 3.5. Convergent validity and composite reliability assessment

Table 7 presents the psychometric properties of the IAT, including factor loadings (ranging from 0.56 to 0.88), AVE (ranging from 0.526 to 0.531), and CR (ranging from 0.869 to 0.885). These values surpass their respective threshold criteria, providing evidence for adequate convergent validity and composite reliability of the IAT. The observed metrics support the instrument’s internal consistency and the extent to which the items effectively represent their intended constructs.

### 3.6. Discriminant validity assessment among sub-constructs

The results of the discriminant validity examination for IAT are presented in Table 8. The square roots of AVE for the three sub-constructs, displayed in italics along the diagonal, exceed all inter-construct correlations. Furthermore, the correlation coefficients between sub-constructs range from 0.628 to 0.712, all falling below the threshold of 0.85. These findings provide evidence for satisfactory discriminant validity among the sub-constructs of the IAT. Consequently, the three-factor solution of the IAT is deemed appropriate and adopted for subsequent analyses in this study.

**Table 8:** Discriminant validity examination for sub-constructs.

Factors	M±SD	WSP	ECP	LCI
WSP	1.89±0.73	*0.726*		
ECP	2.57±0.86	0.628	*0.725*	
LCI	2.68±0.83	0.642	0.712	*0.728*

### 3.7. Criterion validity

Table 9 presents the correlational analyses between internet addiction, as measured by the IAT and its sub-scales, and criterion variables including daily online hours, duration of internet use in years, and academic performance. The results indicate no significant associations between the IAT, including its sub-scales, and either daily online hours or duration of internet use. However, significant and negative correlations are observed between the IAT and academic performance, with the exception of the WSP sub-construct (r = -0.135, p > 0.05).

**Table 9:** Correlations among internet addiction and criterion variables.

Scale	Hours spent online per day	Internet usage years	Academic performance
WSP	0.041	0.015	‒0.135
ECP	0.042	0.138	‒0.307^**^
LCI	0.058	0.071	‒0.231^**^
Total IAT	0.054	0.081	‒0.265^**^

** p < 0.01.

### 3.8. The internet addiction level of the sampled students

Analysis of internet addiction prevalence in the study sample revealed that 39.2% of participants met the criteria for internet addiction, with 37.2% classified as moderate addicts and 1.9% as severe addicts (Table 10). The remaining 60.8% of the sample were categorized as either non-addicted or exhibiting mild addiction symptoms. These findings provide insight into the distribution of internet addiction severity within the studied population and contribute to the growing body of literature on internet addiction prevalence rates.

**Table 10:** The internet addiction level of the sampled students.

Category	Score Range	Frequency (n)	Percentage (%)
Without addiction	0-30	99	9.5
Mild addiction	31-49	535	51.3
Moderate addiction	50-79	388	37.2
Severe addiction	80-100	20	1.9
Total		1042	100%

## 4. Discussion

This study aimed to validate the IAT among Chinese undergraduate students from normal universities. The validation process involved several key steps, including item modification, EFA, CFA, and comprehensive psychometric evaluation. After that, the internet addiction level of the sampled normal undergraduate students was determined using IAT.

Initially, cultural adaptation and translation were undertaken to ensure the IAT’s suitability for the target population. Given the significant changes in internet usage patterns since the IAT’s original development over two decades ago, items 2, 3, 6, 7, 8, 14, and 19 were rephrased based on literature recommendations and contextual considerations [17,20,24,33,34,40]. This adaptation process resulted in good face and content validity, with participants demonstrating clear comprehension of all items. Moreover, the adapted IAT was found to have good psychometric properties, indicating these modifications enhanced the validity and reliability of IAT within the context of Chinese undergraduate internet use.

The EFA results supported a three-factor solution for the IAT, consistent with findings from diverse international studies across various cultural contexts [23,39,44,63,64]. Notably, this three-factor structure appears to be particularly robust within Chinese populations, emerging consistently across validation studies conducted with both undergraduate students and adolescents in Hong Kong and mainland China [12,16,17,20], which suggests a robust underlying structure of IAT within Chinese cultural contexts.

Internal consistency reliability of IAT was high, with Cronbach’s alpha values exceeding 0.70 for all three sub-constructs. This finding is congruent with previous validation studies [11,16,34,39,42,44,65,66]. Moreover, the IAT demonstrated high test-retest reliability over a 14-day interval, consistent with prior reports [24,43,44], which underscores its temporal stability, a crucial psychometric property that enhances its applicability in longitudinal research designs and intervention efficacy studies targeting problematic internet use [15,31]. Such psychometric robustness is particularly valuable in clinical and research contexts where precise, replicable assessments of internet addiction severity are essential for evaluating the effectiveness of preventive strategies and therapeutic interventions [67].

The CFA results confirmed the three-factor model’s good fit to the data, with all items loading significantly onto their respective factors and the factor loadings of the adapted items were more satisfactory than those of previous studies [20,24,62,68–71], indicating that the assessment of internet addiction should keep pace with the times and take into account the characteristics of internet use in specific populations and the impact of sociocultural factors on addictive behavior. Following precedents in the literature, error covariances were correlated for some items rather than deleting them, preserving the IAT’s 20-item structure to maintain cross-cultural equivalence [13,17,19,22–24,35,36,42,62,70,72–74]. Furthermore, the IAT exhibited satisfactory construct validity, convergent validity, composite reliability, and discriminant validity, which is in line with the previous research [9,16,37,43,44].

Criterion validity assessment revealed no significant associations between IAT scores (including subscales) and either daily online hours or duration of internet use. However, significant negative correlations were observed between IAT scores and academic performance, except for the WSP sub-construct. These findings align with previous research [12,22,24,30,66], supporting the IAT’s good criterion validity. The seemingly paradoxical relationship between internet addiction scores, academic performance, and internet usage hours might be because Internet addiction is characterized by loss of control, preoccupation, and continued excessive use despite negative consequences, which may directly impact academic performance through reduced study time, poor concentration, and irregular sleep patterns. However, total internet usage hours alone may not necessarily indicate problematic use, as digital technologies continue to evolve, students increasingly engage in productive online activities, making time-based measurements less reliable indicators of problematic use [12,22].

The detailed profile of internet usage behaviors provides valuable context for interpreting internet addiction scores and aligns with recent literature emphasizing the importance of considering specific online activities in addiction research [75,76]. The high prevalence of watching videos and online chatting reflects broader trends in digital media use among young adults and may have implications for targeted intervention strategies.

In the context of internet addiction prevalence, our findings reveal a substantial rate of 39.2% among the sampled population, with 37.2% classified as moderate addicts and 1.9% as severe addicts, which underscores the significant presence of problematic internet use within the studied population. The observed prevalence is higher than rates reported in previous studies among similar populations [77], which may be due to the annual increase in internet addiction prevalence [1]. The COVID-19 pandemic may have further exacerbated this trend observed over the past two decades [2]. However, the observed prevalence remains lower than rates reported among adolescents and medical students in some countries [14,20,72], suggesting potential demographic or contextual factors influencing addiction rates. While moderate addiction is prevalent, severe cases are relatively rare. This pattern underscores the need for targeted interventions focusing on moderate addiction. The predominance of moderate addiction cases suggests a critical window for intervention before these individuals potentially progress to more severe addiction levels. Notably, 60.8% of participants exhibited either no addiction or mild addiction symptoms, indicating that a majority maintained relatively healthy internet use patterns or experienced only minor issues related to internet use.

### Implications, limitations and recommendations.

This study has both theoretical and practical significance. Theoretically, this study upgraded some of the problematic items found in literature and contributed to the debate of factor solutions of IAT by exploring its structure through an unexplored population before, which provides new evidence for more accurate measurement of internet addiction and the three-factor structure of IAT in the Chinese context. Practically, with established reliability and validity, the IAT can serve as a reliable and valid instrument for identifying and assessing internet addiction among undergraduate students in Chinese normal universities, and the determination of internet addiction levels is helpful for screening potential internet addicts and helps evaluate the effectiveness of intervention strategies. Its psychometric properties support its potential use in clinical settings, educational institutions, and research studies aimed at intervention and prevention strategies.

While these findings provide valuable insights, it is important to consider potential limitations such as self-report bias or the specific characteristics of our sample. This study used self-report measurement, which is affected by the social desirability effect and may cause subjects to underestimate internet addiction and overestimate academic performance. The sample of this study comes from normal university students of one province in China and the potential lack of diversity in the sample may limit the generalizability of research conclusions.

To address the limitations of sample representativeness, it is recommended to conduct a larger-scale study that includes participants from diverse geographical regions within China, which would help ensure the instrument’s applicability across different socio-cultural contexts and enhance its generalizability. Moreover, future studies could explore factors contributing to the development of moderate addiction and investigate the long-term trajectories of internet use patterns in this population. The high prevalence of moderate addiction suggests a need for targeted interventions and preventive measures within this population, which might include awareness programs, digital literacy initiatives, or counseling services tailored to address moderate levels of problematic internet use.

## 5. Conclusions

This study validated a three-factor structure of the IAT among Chinese undergraduate students. The instrument demonstrated satisfactory reliability and validity, indicating its psychometric robustness and suitability for assessing internet addiction in this population. The findings support the IAT’s utility as a reliable measure for future investigations of internet addiction among Chinese undergraduates. Notably, the analysis revealed a high prevalence of moderate internet addiction within the sampled student cohort, underscoring the significance of this issue in the target population and the need for further research and potential interventions.
